# Combined non-invasive neuromodulation using transcranial direct current stimulation, motor imagery and action observation for motor, cognitive and functional recovery in cortico-basal degeneration: a single case study

**DOI:** 10.17179/excli2024-7027

**Published:** 2024-05-06

**Authors:** Juan Pablo Romero, Alexis Martínez-Benito, David de Noreña, Alfonso Hurtado-Martínez, Francisco José Sánchez-Cuesta, Yeray González-Zamorano, Marcos Moreno-Verdú

**Affiliations:** 1 Brain Injury and Movement Disorders Neurorehabilitation Group (GINDAT), Francisco de Vitoria University, Pozuelo de Alarcón, 28223, Spain; 2 Facultad de Ciencias Experimentales, Universidad Francisco de Vitoria, Pozuelo de Alarcón, 28223, Spain; 3 Brain Damage Unit, Beata María Ana Hospital, Madrid, 28007, Spain; 4 Cognitive Neuroscience, Pain and Rehabilitation Research Group (NECODOR), Faculty of Health Sciences, Rey Juan Carlos University, Madrid, Spain; 5 Departamento de Fisioterapia, Centro Superior de Estudios Universitarios La Salle, Universidad Autónoma de Madrid, Madrid, Spain; 6 Escuela Internacional de Doctorado, Department of Physical Therapy, Occupational Therapy, Rehabilitation and Physical Medicine, Universidad Rey Juan Carlos, 28933 Alcorcón, Spain; 7 Department of Physical Therapy, Occupational Therapy, Rehabilitation and Physical Medicine, Universidad Rey Juan Carlos, 28933 Alcorcón, Spain; 8 Brain, Action and Skill Laboratory (BAS-Lab), Institute of Neuroscience (Cognition and Systems Division), UC Louvain, 1200 Woluwe-Saint-Laimbert, Belgium

**Keywords:** corticobasal degeneration, non-invasive neuromodulation, transcranial direct current stimulation, motor imagery, action observation

## Abstract

This case report presents a comprehensive assessment and therapeutic intervention using non-invasive motor cortex neuromodulation for a 70-year-old female patient diagnosed with corticobasal degeneration (CBD). The study followed the CARE guidelines. The patient meets the criteria for probable CBD, with neuroimaging evidence of exclusively cortical impairment. The patient underwent a non-invasive neuromodulation protocol involving transcranial direct current stimulation (tDCS) and action observation plus motor imagery (AO+MI). The neuromodulation protocol comprised 20 sessions involving tDCS over the primary motor cortex and combined AO+MI. Anodal tDCS was delivered a 2 mA excitatory current for 20 minutes. AO+MI focused on lower limb movements, progressing over four weeks with video observation and gradual execution, both weekly and monthly. The neuromodulation techniques were delivered online (i.e. applied simultaneously in each session). Outcome measures were obtained at baseline, post-intervention and follow-up (1 month later), and included motor (lower limb), cognitive/neuropsychological and functional assessments. Walking speed improvements were not observed, but balance (Berg Balance Scale) and functional strength (Five Times Sit-to-Stand Test) improved post-treatment. Long-term enhancements in attentional set-shifting, inhibitory control, verbal attentional span, and working memory were found. There was neurophysiological evidence of diminished intracortical inhibition. Functional changes included worsening in Cortico Basal Ganglia Functional Scale score. Emotional well-being and general health (SF-36) increased immediately after treatment but were not sustained, while Falls Efficacy Scale International showed only long-term improvement. The findings suggest potential benefits of the presented neuromodulation protocol for CBD patients, highlighting multifaceted outcomes in motor, cognitive, and functional domains.

## Introduction

Corticobasal Degeneration (CBD) is a rare progressive neurodegenerative tauopathy that typically presents with asymmetric rigidity, apraxia, dystonia, myoclonus, alien limb phenomenon, parkinsonism, and cortical sensory loss as well as behavioral and cognitive impairments (Armstrong et al., 2013[4]; Constantinides et al., 2019[17]; Lyons et al., 2023[37]). Not all the patients present the same symptoms as the disease can be manifested through different clinical phenotypes or syndromes (Cortico-basal syndrome (CBS), frontal behavioral-spatial syndrome (FBS), non-fluent agrammatic primary progressive aphasia syndrome (naPPA) and progressive supranuclear palsy (PSP)) (Alexander et al., 2014[2]). The diagnosis requires insidious onset and gradual progression for at least 1 year, age onset ≥50 years, no similar family history or known tau mutations, and a clinical phenotype of probable CBS or either FBS or naPPA with at least 1 CBS feature (Koga et al., 2022[31]).

Regarding treatment options, no disease-modifying treatment has been approved for CBD (Bluett et al., 2021[9]). Although levodopa can be used to treat parkinsonian symptoms accompanying some of the phenotypes of the disease, its efficacy is not complete. Due to the rapid progression of CBD with an average survival time of 6.5 years (Lyons et al., 2023[37]), symptomatic treatment is the only possible approach. Among symptomatic treatment for motor and cognitive symptoms, physical and cognitive rehabilitation have been used to provide care and support to patients during this catastrophic disease. Physical therapy, based on repetitive tasks, has been previously used in these patients with slight improvements of functional domains (Kawahira et al., 2009[29]; Steffen et al., 2014[57]; Fusco et al., 2018[23]). These types of therapies, although relatively effective, are known to require long term protocols that are often limited in patients with rapid disease progression.

Neurophysiological and neuroimaging studies in CBD have shown asymmetric abnormalities in regional glucose metabolism, cortical excitability and transcortical inhibition (Murgai and Jog, 2018[41]). These changes seem to be correlated with motor manifestations such as limb apraxia (Burrell et al., 2014[12]).

Transcranial Direct Current Stimulation (tDCS) is an exogenous non-invasive neuromodulation technique oriented to modify cortical excitability (Chase et al., 2020[15]). It is based on the delivery of a relatively weak continuous current over the brain cortex through small electrodes placed on the scalp (Nitsche and Paulus, 2000[43]; Lang et al., 2005[33]). Depending on whether the active electrode is in the anode or cathode, it increases or decreases cortical excitability, respectively (Fregni et al., 2021[22]). tDCS protocols have been previously used in other neurological conditions to enhance cognitive and motor domains (Lefaucheur et al., 2017[34]). There is evidence that this therapy can be combined with conventional physical rehabilitation to increase its effectiveness (Navarro-López et al., 2021[42]). The use of this technique has been previously reported effective in CBD to enhance apraxia and action naming (Bianchi et al., 2015[8]; Manenti et al., 2015[38]). However, its effects on other motor, cognitive and functional variables are still unknown.

On the other hand, endogenous non-invasive neuromodulation techniques such as Motor Imagery (MI) and Action Observation (AO) training have also been demonstrated to enhance cortical activation (Leocani et al., 2012[35]) and have been used in combination with physical rehabilitation to enhance its effectiveness (Mulder, 2007[39]). MI is defined as a cognitive and dynamic state during which representations of a given motor act are internally rehearsed in working memory without any overt motor output (Decety, 1996[19]), whereas AO training is considered as the internal representation of a set of movements evoked by the observer during visualization of the movement (Buccino, 2014[11]). These action simulation approaches activate a brain network that substantially overlaps with action execution, and their combination has shown to synergically increase corticospinal excitability (Wright et al., 2014[66]). MI and AO have been widely used for motor rehabilitation in multiple neurological pathologies (Caligiore et al., 2017[14]; Guerra et al., 2017[27]; Opsommer et al., 2020[46]; Suso-Martí et al., 2020[58]; Cuenca-Martínez et al., 2022[18]). In combination with physical exercise, they have been effective in improving postural instability and gait disturbances in people with Parkinson's disease (Tremblay et al., 2008[60]; Sarasso et al., 2021[54]), but have never been used in CBD. 

Due to the inexistence of effective therapy for CBD and due to the limited therapeutical efficacy of conventional physical rehabilitation on a rapidly progressive neurodegenerative disease, in this study we aimed to assess the motor, cognitive, and functional effects of a protocol combining tDCS, AO, and MI to enhance physical therapy in a 71-year-old woman diagnosed with CBD. The primary objective was to design a feasible and personalized protocol for the combined application of these techniques focused on motor and functional performance. Secondarily, we sought to investigate whether the observed effects were associated with improvements in quality of life and neuropsychological outcomes.

## Material and Methods

We present a case report study following the CARE checklist (Riley et al., 2017[52]). The study was approved by an ethics committee in Madrid and patient provided informed consent following the principles of the Declaration of Helsinki of 1964, updated to its latest version in 2013 (World Medical Association 2013[65]).

### Case presentation

A 71-year-old female patient with no relevant previous medical history and no family history of neurological diseases presented with a two-year history of postural instability and multiple falls that have progressed to confine her to a wheelchair most of the day. During the physical assessment, muscle strength was found to be normal, but hyperreflexia, predominantly in the lower limbs, was observed. Sensitivity, cranial nerves, and cerebellar function were normal. When testing lower limb mobility, she had difficulty performing tasks such as raising the left toe or tapping her foot, spontaneously noting that her leg did not respond to her commands and produced unintended spontaneous movements, leading to a diagnosis of alien limb syndrome. Over time, the impairment progressed to affect the upper left arm with occasional dystonic posture. An extensive neuropsychological study revealed alterations in executive functions, attentional components, and executive memory. The patient was aware of her deficits and displayed a depressive mood.

Brain and neuroaxis magnetic resonance imaging (MRI) showed no abnormalities. An electroencephalogram (EEG) and electromyography of the lower limbs produced normal results. Functional assessments included a normal 185 MBq FP-CIT (ioflupano) scan (DAT scan) in the early stages of her disease. This test was not repeated. On the other hand, a cerebral 18-Fluorodeoxyglucose PET scan (FDG-PET) revealed right frontal hypoactivity. The FDG-PET was repeated one year later, revealing worsened hypoactivity extending partly to the left hemisphere. Extensive genetic testing was performed and MAPT mutation was discarded along with other genetic causes of dementia or movement disorders. CBD was suspected.

Empirical therapy with levodopa was initiated at the onset due to slight left sided parkinsonism, reaching 900 milligrams per day with limited improvement and subsequent discontinuation without evident deterioration. The patient underwent conventional rehabilitation, including two weekly sessions of physical therapy from the onset of the condition, with limited or no improvement. 

This particular patient was chosen for the study because her symptoms meet the criteria for probable CBD, characterized by asymmetric limb akinesia, limb dystonia, limb apraxia, alien limb phenomena, and the absence of MAPT mutations (Armstrong et al., 2013[4]). However, her neuroimaging findings were atypical, yet plausible; the DAT scan was normal, and the PET-CT scan did not show basal ganglia or thalamic impairment, thus not indicating subcortical involvement (Cilia et al., 2011[16]; Pardini et al., 2019[49]). This presents a unique opportunity to explore the potential benefits of therapeutic approaches that aim to modulate cortical excitability.

The neuromodulation protocol presented in this report was specifically designed for this patient with the goal of enhancing right frontal cortical activation due to the findings on neuroimaging functional tests.

### Neuromodulation protocol

The protocol consisted of 20 sessions of tDCS+AO+MI (Monday to Friday) while continued receiving her regular conventional physical therapy two days per week. Non-invasive neuromodulation techniques were combined and always applied “online” (i.e. AO+MI and tDCS were delivered simultaneously in each session).

### tDCS

Each session of tDCS consisted in 20 minutes of a 2 mA direct galvanic excitatory current with 30 seconds of ramp-up and 30 seconds of ramp-down over the primary motor cortex (M1) using a saline-soak pair of surface sponge electrodes (35 cm2). The anode electrode was placed over the right M1 (C3 position according to the EEG 10/20 system), and the cathode electrode over the contralateral supraorbital area (Fp2). A Starstim tDCS^® ^stimulator (Neuroelectrics, Barcelona, Spain) was used.

### AO + MI

**Figure 1:**
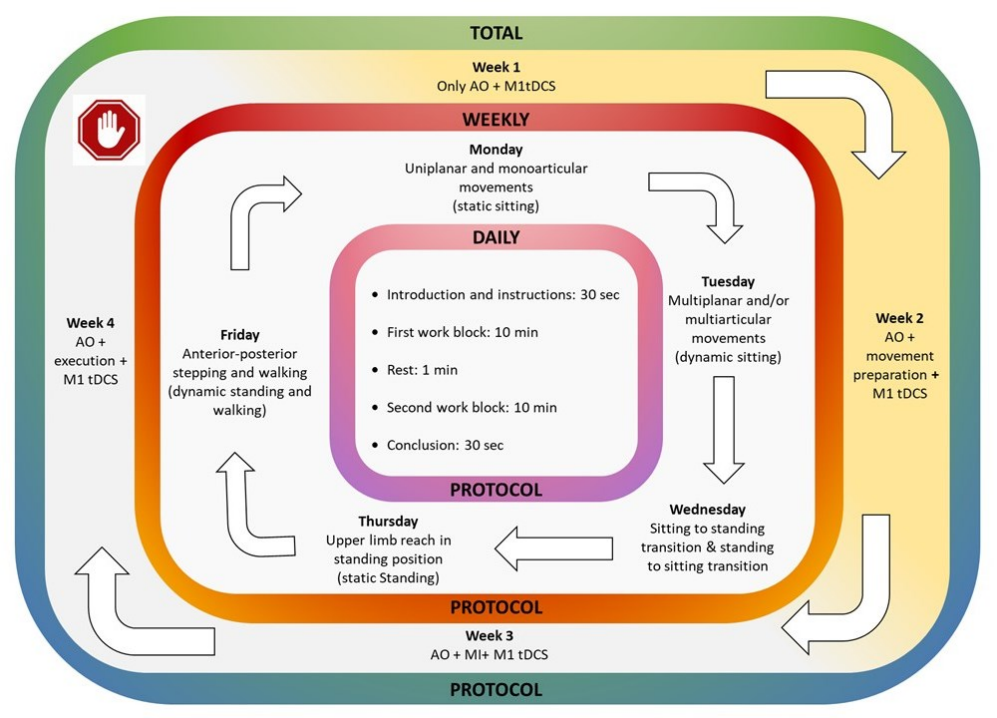
The four-week intervention protocol. The patient received 20 sessions in four weeks from Monday to Friday. Mental representation techniques followed a graded progression: Only Action Observation (first week); Action Observation + Movement Preparation (second week); Action Observation + Motor Imagery (third week); Action Observation + Movement Execution (fourth week). Different movements were assigned to each day of the week and were performed according to the corresponding type of representation technique. Neuromodulation therapy through transcranial direct current stimulation was applied every session. The protocol duration was 22 minutes divided in “Introduction and instructions” (30 seconds), “First work block” (10 minutes of the corresponding daily protocol), “Rest” (1 minute), “Second work block” (10 minutes continuing the daily protocol), and “Conclusions” (30 seconds). AO: Action Observation; M1: primary motor cortex; MI: Motor Imagery; tDCS: Transcranial Direct Current Stimulation

As the main complaint of this patient and the initial symptom was lower limb alteration, limiting walking and standing even with normal lower limbs strength, the AO+MI protocol was centered on lower extremities movements, encompassing both static and dynamic sitting, the transition from sitting to standing and vice versa, as well as gait. The intervention was delivered with graded weekly progression as shown in Figure 1(Fig. 1). Monthly progression was achieved as follows: In the first week, the intervention commenced with exclusive AO sessions. These AO sessions involved the patient watching videos of a physiotherapist (A.M.B.) demonstrating various movements and actions. The second week introduced the preparation for movement component alongside the ongoing AO. The patient was instructed to “prepare for the movement” but not to perform it or imagine it. During the third week, MI was incorporated, continuing the AO exposure, and therefore AO+MI was used. The patient was instructed to “try to imagine the movement at the same time you see it”, incorporating both visual and kinesthetic components. The final week integrated movement execution with AO. The patient was instructed to try to execute the movements at a similar pace as it was shown in the videos. Because of motor deficits, the speed of the movements showed in the videos was slightly adapted when the patient could not follow the same pace as the model. 

Each week, the intervention was structured to follow a progression from uniplanar/monoarticular movements (see supplementary information; day 1; Supplementary Figure 1a) and multiplanar/multiarticular movements (day 2; Supplementary Figure 1b) while sitting, to transfers (day 3; Supplementary Figure 1c) and postural stability components through weight shifting (day 4; Supplementary Figure 1d), finishing always with gait practice (day 5; Supplementary Figure 1e). Each daily session commenced with a 30-second introduction and instructions, followed by two 10-minute working blocks separated by a 1-minute rest period. The intervention concluded with a 30-second final summary.

### Outcome measures

Outcome measures were assessed by two trained physical therapists and one neuropsychologist at three different time points: pre-intervention (day 1, measurement A), two days after the intervention was finished (day 32, measurement B) and after one month (day 60, measurement C). Between measurements B and C, the patient only received conventional physical therapy. Three tests were used to evaluate motor capacity as primary outcome measures. Secondary outcomes included functional, neuropsychological, and neurophysiological evaluations.

### Motor variables

The 10-Meter Walk Test (10-MWT) was used to assess gait speed using a walker as assistive device. The patient was instructed to walk 10 meters at her normal pace. The 10 meters were divided into three parts by marks on the floor: 2 meters at the beginning, 6 meters in the middle and 2 meters at the end. The time taken to walk through the middle part was recorded to calculate gait speed (meters per second), considering that the first and last parts were for acceleration and deceleration, respectively (Watson, 2002[63]; Steffen and Seney, 2008[56]).

The Berg Balance Scale (BBS) was used to assess balance and risk of falling. The BBS consists of a series of 14 different-balance related tasks designed to simulate everyday activities and range in difficulty from sitting and standing to activities such as reaching and turning around. Higher scores indicate better performance (Berg et al., 1995[7]; Usuda et al., 1998[61]; Blum and Korner-Bitensky, 2008[10]; Downs et al., 2013[20]).

The 5-Times Sit-to-Stand Test (5TSST) was used to evaluated functional strength, as the time needed to stand up fully from the seated position and sit back again five times as fast as possible (Duncan et al., 2011[21]; Muñoz-Bermejo et al., 2021[40]).

### Cognitive variables

Digits and Symbol Search subtests, which belong to the Wechsler Adults Intelligence Scale, fourth edition (WAIS-IV), the Trail Making Test and the STROOP test were utilized. 

WAIS-IV's Digit subtests were administered to evaluate working memory and attentional span (phonological loop). Both subtests require the participant to learn and recall number sequences in different orders. The Symbol Search task was used to evaluate visual perception, working memory and processing speed, as it involves identifying a symbol's duplicate from among a list (Wechsler, 2012[64]).

The Trail Making Test (TMT) is a test in which the participant must draw a line, connecting a set of numbered dots in the correct order, as fast as possible. Part A is a number sequence, while part B is an alternating number-letter sequence. They measure processing speed, visual perception, eye-to-hand coordination and mental flexibility, attentional set shifting and inhibition, respectively (Tombaugh, 2004[59]). 

The STROOP test is comprised of three parts: word, color, and word-color. The first two parts both measure visual perception and attention, processing speed, but also the visual routes of language in the form of reading and denomination. The third part evaluates the more executive-leaning processes of attention: attentional control, inhibition of an overlearned skill (reading) and interference control (Scarpina and Tagini, 2017[55]).

For neuropsychological tests, scores were normalized with the help of Spanish-validated scales (except WAIS-IV's symbol search). Scales for the TMT, both inverse and direct digits and STROOP test can be found in Peña-Casanova et al. (2009[50]), and scores for the WAIS-IV symbol search test were standardized using the WAIS' built-in scales. Age corrections were applied on every scale. 

### Functional variables

The Cortical Basal Ganglia Functional Scale (CBFS) was used to evaluate the functional impact of CBD. The CBFS is a self-report questionnaire that consists of 14 questions related to motor aspects in daily living, and 17 questions related to non-motor aspects in daily living. Higher scores indicate more severe impact (Lang et al., 2020[32]).

The Falls Efficacy Scale International (FES-I) questionnaire is a self-report assessment tool used to measure the perceived level of confidence in the ability to avoid falling during various activities of daily life, with lower values suggesting higher self-confidence (Lomas-Vega et al., 2012[36]). 

The Short Form Health Survey-36 (SF-36) was used to measure health-related quality-of-life. The SF-36 is a 36-item questionnaire where the person rates, using a Likert type scale, 8 domains of quality of life: physical functioning, role physical, bodily pain, general health, vitality, social functioning, role emotional, and mental health. Higher scores indicate better quality-of-life (Alonso et al., 1995[3]).

### Neurophysiological variables

The only neurophysiological measure was the Cortical Silent Period (CSP), collected using single-pulse Transcranial Magnetic Stimulation (TMS). Electromyography (EMG) recording coupled to TMS was collected using 9 mm diameter Ag-AgCl surface electrodes placed on the first dorsal interosseus muscle. EMG measurements were amplified 1000 times, filtered with a band pass of 20 Hz - 2.5 kHz using a Digitimer D440-2 amplifier (Digitimer Ltd., UK) and digitized with a CED Micro 1401-3 (Cambridge Electronic Design, UK) (Groppa et al., 2012[26]). All EMG data were pre-processed and analyzed using Signal software, version 6 (Cambridge Electronic Design, UK). For CSP, ten consecutive single-pulse TMS at 130 % RMT were applied during weak voluntary contraction (15-20 % of the participant's maximal voluntary contraction) of the FDI. The MEP-onset until return of voluntary EMG activity time (in milliseconds) was measured as CSP for each hemisphere separately as an average of all 10 measurements (Hupfeld et al., 2020[28]).

### Data analysis

This case report did not include appropriately spaced subsequent interventions to compare with, and therefore no formal statistical analyses were implemented. Results are reported as changes from baseline to post-intervention and to follow-up for each outcome measure. 

## Results

The main results of this case report are shown in Table 1(Tab. 1).

### Tolerance and adherence

There was a good tolerance to the proposed protocol and the adherence to all the sessions was complete. No adverse effects were reported.

### Motor capacity

No significant increase in walking speed was observed either after treatment or during the follow-up period. Improvements were observed in balance and functional strength after treatment, as indicated by the BBS (16 to 23 points) and the 5TSTS (83 to 52.89 seconds). Improvements in postural control were partially sustained in the long term (5TSTS: 83 to 67 seconds), while changes in balance did not persist.

### Cognitive variables

Regarding cognitive and neuropsychological variables, a sustained long-term enhancement in attentional set-shifting and inhibitory control (TMT-B: +0.33σ post-treatment, +0.66σ follow-up) was found. Additionally, a persistent improvement in verbal attentional span during the follow-up, relative to baseline, was evidenced (Direct Digits +1σ in the follow-up). A long-lasting post treatment improvement in working memory was observed, characterized by an increase of 1σ in Inverse Digits scores both post-treatment and during the follow-up. Furthermore, there was an enduring long-term rise in color denomination (STROOP-C +0.66σ in the follow-up). STROOP interference measures showed a significant increase in score after treatment.

### Functional variables

When comparing pre-treatment and post-treatment evaluations for CBFS, no significant difference was observed in total scores (23 and 22 points), but a negative trend was noticeable during the follow-up evaluation (total score of 30 points).

Emotional well-being and general health dimensions of the SF-36 showed immediate increases after treatment. However, these improvements were not sustained during the follow-up period. The FES-I did not exhibit an immediate increase in scores after treatment, but a noticeable long-term improvement was observed during the follow-up.

### Neurophysiological variables (CSP)

In the most affected hemisphere (right hemisphere) the motor evoked response was not obtained after stimulating withing the ranges recommended in the safety recommended guidelines (70 % output). CSP increases from baseline to post intervention.

## Discussion

A multidimensional protocol based on tDCS, AO and MI was applied to a CBD-affected woman with a one-month follow-up, obtaining motor, cognitive, functional and neurophysiological improvements. 

Literature examining the effects of AO therapy, MI and tDCS on the motor and cognitive alterations present in CBD is scarce. However, by drawing upon research in similar neurodegenerative illnesses such as Parkinson's disease, many conclusions can be drawn from this case.

Multi-session and long-term therapeutic programs based on either AO or MI can improve general motor abilities in Parkinson's patients, particularly so when the disease is at its early stages (Caligiore et al., 2017[14]). The techniques are used along physical therapy, and the tasks have high ecological validity (Caligiore et al., 2017[14]), and MI is a recommended method for neurorehabilitation that has been used to improve gait and balance in this disease (Abraham et al., 2021[1]). tDCS has also proved its efficacy in significantly improving mobility, balance, gait velocity and fall reduction in Parkinson's disease (Orrù et al., 2019[47]). 

The evidenced alterations in executive functions, attentional components, and operative memory align with the cognitive profile typically associated with CBD (Oliveira et al., 2017[45]). Recent studies have shown that AO training, particularly when paired with another task, can improve working memory and focused and sustained attention (Caligiore et al., 2019[13]). TDCS has also shown promise in the tackling of cognitive variables: anodal stimulation over M1, as used in this study, can improve inhibitory control and action selection in healthy subjects (Bashir et al., 2022[5]; Rizvi et al., 2023[53]). When applied over the dorsolateral prefrontal cortex, it has been shown to improve reasoning, executive function, attention and working memory in patients with neurodegenerative diseases such as multiple sclerosis or Parkinson, and even psychiatric disorders (Begemann et al., 2020[6]; Gholami et al., 2021[24]).

Given the aforementioned evidence, it is reasonable to assume that a combined application of all three techniques could account for the post-intervention improvement in balance, gait speed and functional strength, even if only some of the changes persisted over time. Said evidence is also concurrent with the long-term enhancement in attentional set-shifting, inhibitory control and working memory -some of the most prominent executive dysfunctions associated with CBD as well as the increase in verbal attentional span during the follow-up (Oliveira et al., 2017[45]).

The mechanisms underlying the effects of our protocol are likely complex, involving both cortical and subcortical elements. On one hand, our protocol has been shown to modify CSP, decreasing its duration, which is consistent with previous reports. Anodal tDCS of the primary motor cortex has been found to reduce intracortical inhibition (Nitsche et al., 2005[44]). Intracortical inhibition is a neural mechanism that helps focus excitatory drive and is believed to be mediated by GABA_B receptors (Poston et al., 2012[51]). Our protocol may have facilitated the activation of a wider cortical area as the intracortical inhibition decreased. On the other hand, during motor imagery, the coupling of a feedforward network from the PMC to the SMA and of a feedback network from M1 to the PMC has been shown to be even stronger than during motor execution (Kim et al., 2018[30]). AO has also been demonstrated in animal studies to effectively activate motor neurons in the M1 (Vigneswaran et al., 2013[62]). These multiple mechanisms coinciding in M1 may have potentiated each other to achieve the motor and cognitive improvements observed. This is in line with the latest descriptions of the primary motor cortex as suggest that M1 is interspersed with a system for the planning of whole-body integral actions, the somato-cognitive action network (SCAN) (Gordon et al., 2023[25]).

### Limitations

The main limitation of this study is that it is conceptualized as a single case report, and therefore the evidence provided is preliminary and exploratory. Although our intervention protocol was long enough to generate changes in motor, cognitive or functional outcomes, a longer protocol in terms of number of sessions, or a training with higher intensity in terms of repetitions per session, might have been able to increase effect sizes, in the context of a chronic and progressive disorder. The lack of a second or third period(s) without any treatment or sham treatment prevented from performing any formal statistical analysis, although taking the personal situation of this patient into account, we considered it unethical and inappropriate. Absence of neuroimaging data pre-post intervention impeded further insights into the neurobiological mechanisms of the intervention delivered.

## Conclusions

The observed quantitative enhancements in motor and cognitive function scores imply a potentially promising role for a compound intervention involving AO, MI and tDCS in mitigating the motor and cognitive alterations associated with CBD. This outcome underscores the need for further research investigating the interactions and potential combinations of these therapeutic modalities in the context of CBD, as well as their comparative effectiveness in other neurodegenerative diseases such as Parkinson's. This line of inquiry not only contributes to our understanding of the therapeutic landscape for CBD but also presents opportunities for developing targeted interventions that may have broader applications in the field of neurodegenerative disorders.

## Declaration

### Acknowledgments

The authors extend their sincere gratitude to the staff at the Texum Clinic in Madrid for diligently maintaining the conventional therapy for the patient involved in this study. The authors would also like to thank Beatriz Madroñero Miguel for her assistance in creating the figures. This work was supported by the Spanish Ministry of Science and Innovation grant (PID2020-113222RBC21/AEI/10.13039/501100011033). 

### Conflict of interest

The authors declare that they have no conflict of interest, because the beforementioned institution did not have any role in the study design, data collection and analysis, decision to publish or preparation of the manuscript.

## Supplementary Material

Supplementary information

## Figures and Tables

**Table 1:**
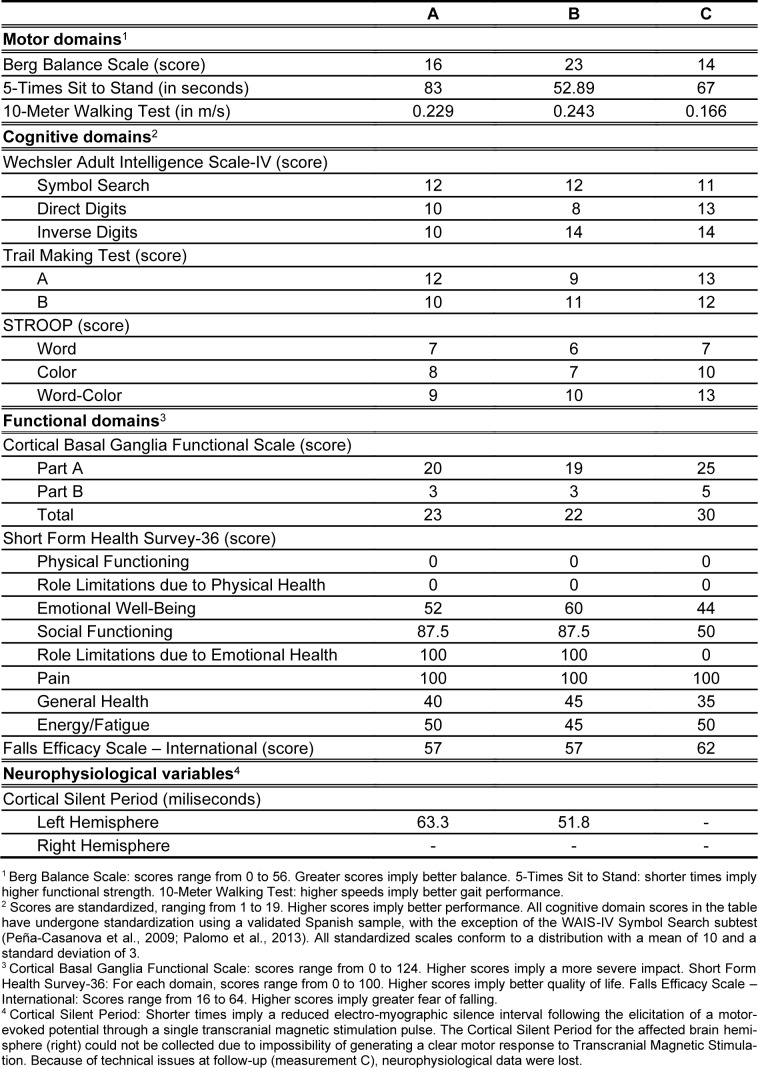
Outcome measures pertaining to each domain evaluation. Score columns A to C correspond to each of the 3 evaluations: baseline, post-intervention and follow-up.
