# Supplementary material for: Combined non-invasive neuromodulation using transcranial direct current stimulation, motor imagery and action observation for motor, cognitive and functional recovery in cortico-basal degeneration: a single case study
Source: EXCLI J. 2024 May 6;23:714–26. doi: 10.17179/excli2024-7027 (PMC11180953; doi:10.17179/excli2024-7027)
Supplement: Supplementary information [file EXCLI-23-714-s-001.pdf]

EXCLI Journal 2024;23:714-726 – ISSN 1611-2156
Received: January 26, 2024, accepted: April 24, 2024, published: May 06, 2024

Supplementary information to:

Case report:

COMBINED NON-INVASIVE NEUROMODULATION USING TRANSCRANIAL DIRECT CURRENT STIMULATION, MOTOR IMAGERY AND ACTION OBSERVATION FOR MOTOR, COGNITIVE AND FUNCTIONAL RECOVERY IN CORTICO-BASAL DEGENERATION: A SINGLE CASE STUDY

Juan Pablo Romero[1,2,3,4], Alexis Martínez-Benito[1,5], David de Noreña[3], Alfonso Hurtado-Martínez[1,2], Francisco José Sánchez-Cuesta[1,2], Yeray González-Zamorano[1,4,6,7,*], Marcos Moreno-Verdú[1,8]

1 Brain Injury and Movement Disorders Neurorehabilitation Group (GINDAT), Francisco de Vitoria University, Pozuelo de Alarcón, 28223, Spain.
2 Facultad de Ciencias Experimentales, Universidad Francisco de Vitoria, Pozuelo de Alarcón, 28223, Spain
3 Brain Damage Unit, Beata María Ana Hospital, Madrid, 28007, Spain
4 Cognitive Neuroscience, Pain and Rehabilitation Research Group (NECODOR), Faculty of Health Sciences, Rey Juan Carlos University, Madrid, Spain
5 Departamento de Fisioterapia, Centro Superior de Estudios Universitarios La Salle, Universidad Autónoma de Madrid, Madrid, Spain
6 Escuela Internacional de Doctorado, Department of Physical Therapy, Occupational Therapy, Rehabilitation and Physical Medicine, Universidad Rey Juan Carlos, 28933 Alcorcón, Spain
7 Department of Physical Therapy, Occupational Therapy, Rehabilitation and Physical Medicine, Universidad Rey Juan Carlos, 28933 Alcorcón, Spain
8 Brain, Action and Skill Laboratory (BAS-Lab), Institute of Neuroscience (Cognition and Systems Division), UC Louvain, 1200 Woluwe-Saint-Laimbert, Belgium

* **Corresponding author:** Yeray González-Zamorano, Department of Physical Therapy, Occupational Therapy, Rehabilitation and Physical Medicine, Universidad Rey Juan Carlos, Atenas Avenue s/n., 28933 Alcorcón, Spain. Tel: +34 689 10 53 57; E-mail: yeray.gonzalez@urjc.es

https://dx.doi.org/10.17179/excli2024-7027

This is an Open Access article distributed under the terms of the Creative Commons Attribution License (http://creativecommons.org/licenses/by/4.0/).

EXCLI Journal 2024;23:714-726 – ISSN 1611-2156
Received: January 26, 2024, accepted: April 24, 2024, published: May 06, 2024

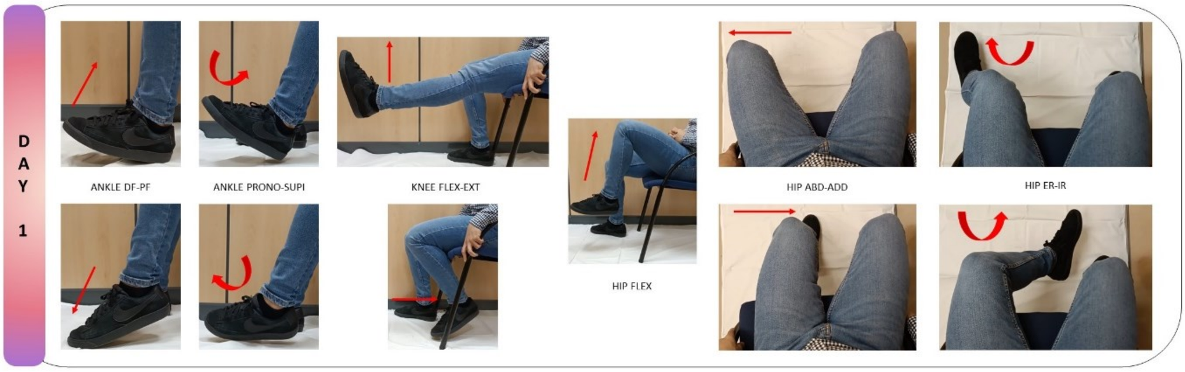

Supplementary Figure 1: The weekly intervention protocol based on graded progression of mental representation techniques.

a. Representation of the Monday treatment protocol: Uniplanar and monoarticular movements (static sitting). ABD: abduction; ADD: Adduction; DF: Dorsal Flexion; FLEX: Flexion; ER: External Rotation; EXT: Extension; IR: Internal Rotation; PF: Plantar Flexion; PRONO: Pronation; SUPI: Supination

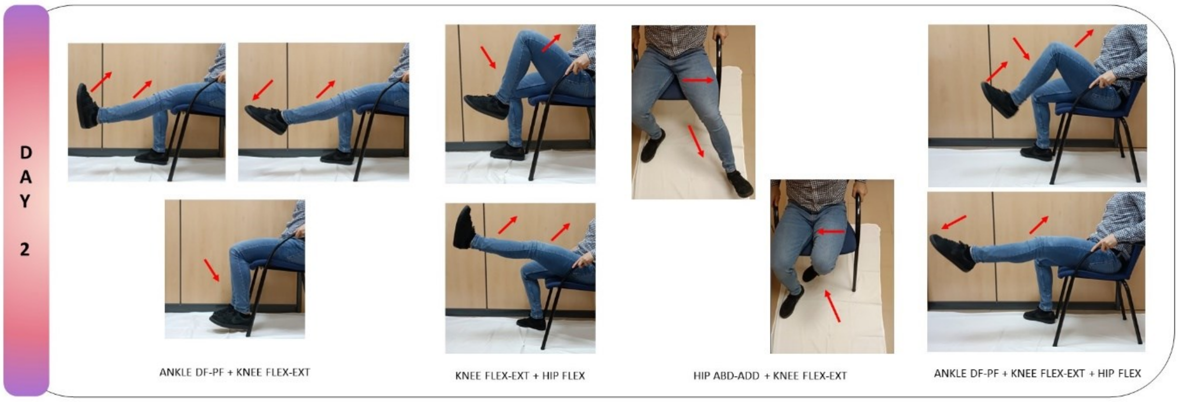

b. Representation of the Tuesday treatment protocol: Multiplanar and/or multiarticular movements (dynamic sitting). ABD: abduction; ADD: Adduction; DF: Dorsal Flexion; FLEX: Flexion; EXT: Extension; PF: Plantar Flexion

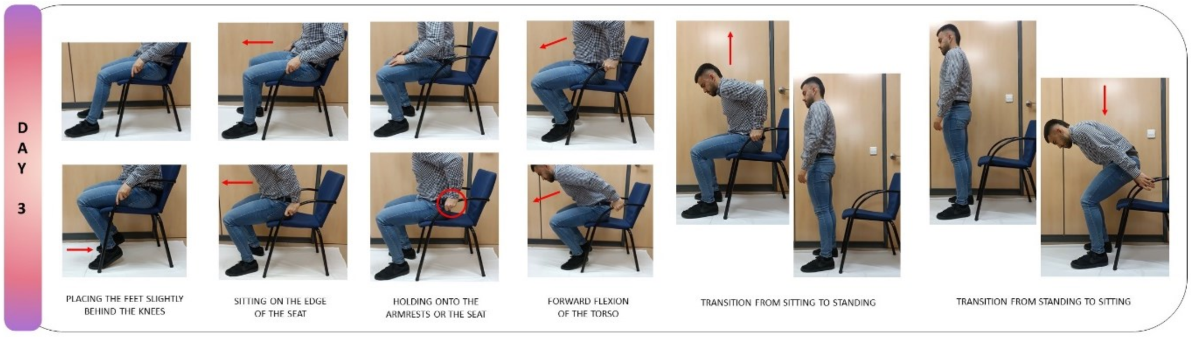

c. Representation of the Wednesday treatment protocol: Sitting to standing transition and standing to sitting transition

EXCLI Journal 2024;23:714-726 – ISSN 1611-2156
Received: January 26, 2024, accepted: April 24, 2024, published: May 06, 2024

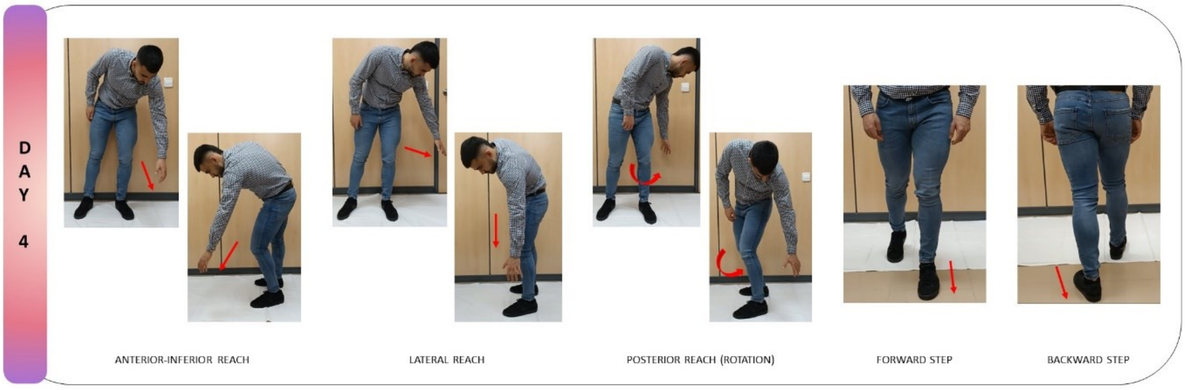

d. Representation of the Thursday treatment protocol: Upper limb reach in standing position (static standing)

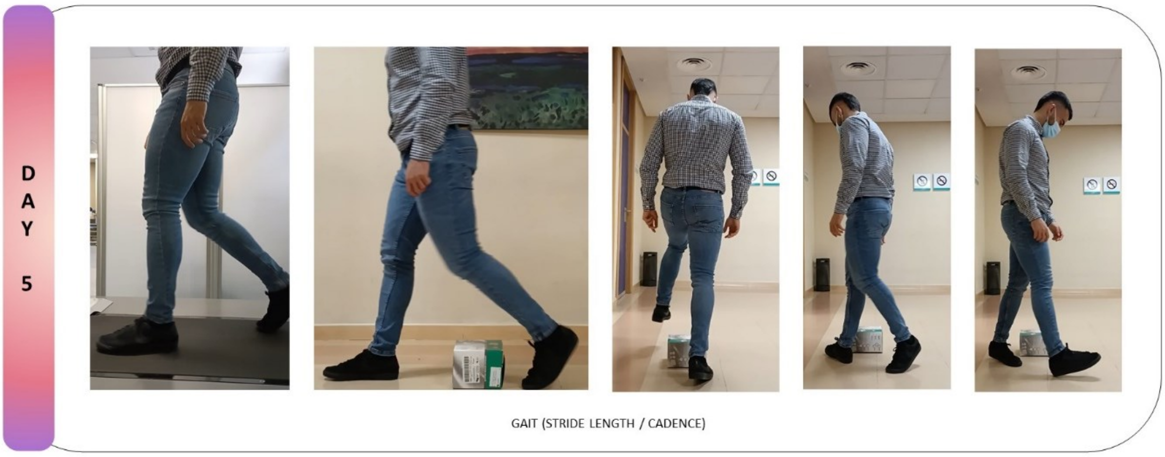

e. Representation of the Friday treatment protocol: Anterior-posterior stepping and walking (dynamic standing and walking)
